# Laser Therapy Versus Traditional Scaling and Root Planing: A Comparative Review

**DOI:** 10.7759/cureus.61997

**Published:** 2024-06-09

**Authors:** Abdulaziz M Altalhi, Luluwah N AlNajdi, Salman G Al-Harbi, Asma M Aldohailan, Jalal Y Al-Ghadeer, Jafar I Al-Bahrani, Zainab J Al-Gahnem, Asma H Alenezi, Ahmed Al-Majid

**Affiliations:** 1 Periodontics, Ministry of Health, Riyadh, SAU; 2 Dentistry, King Salman Armed Forces Hospital, Tabuk, SAU; 3 Dentistry, Ministry of Health, Tabuk, SAU; 4 Dentistry, Al-Muhaidib Dental Clinic, Al-Khobar, SAU; 5 Dentistry, Ministry of Health, Al-Ahsa, SAU; 6 Dentistry, Al-Ahsa Health Cluster, Al-Ahsa, SAU; 7 Dentistry, University of Hail, Hail, SAU; 8 Periodontology, Ministry of Health, Al-Ahsa, SAU

**Keywords:** clinical outcomes laser periodontal, periodontal treatment laser comparison, diode laser periodontal therapy, co2 laser periodontal tretment, periodontitis

## Abstract

Scaling and root planing (SRP) removes bacterial plaque, calculus, and associated microorganisms from the surface of the tooth and the surrounding soft tissue. While complete eradication of pathogenic microbes is impossible, gross removal can lower the microbial load, which in turn reduces inflammation and contributes to positive clinical outcomes. Instrumentation in limited-access anatomic areas like furcation, grooves, deep pockets, concavities, and distal molar areas is technically demanding with traditional mechanical debridement. However, emerging advanced systems such as lasers with bactericidal and detoxification effects offer the benefit of reaching limited-access areas that traditional SRP cannot reach.

A digitized search was done on PubMed, Embase, Medline, and Google Scholar using the keywords “lasers”, “periodontal therapy”, “scaling”, and “root planing”. Upon screening and reviewing, the shortlisted articles comprised narrative reviews, systematic reviews, randomized controlled trials, comparative studies, split-mouth studies, case series, and reports of non-surgical laser therapy and lasers as an adjunct to SRP. This review presents a comprehensive comparative evaluation of different laser modalities utilized in non-surgical periodontal treatment in contrast to standard SRP, rather than an exhaustive article review. It delves into the history and development of lasers, highlighting key advancements made in the realm of periodontics and dental care. This review includes an elucidation of the theory behind laser operation, along with an exploration of its intrinsic characteristics and breakdown of the various types of lasers that are currently available, and an examination of existing literature in both present and historical contexts regarding the comparison of various types of lasers with traditional periodontal treatment.

## Introduction and background

Periodontitis is a chronic disease of an inflammatory nature. Although it has multiple risk factors that involve the periodontium, the primary risk factor is virulent periodontal microorganisms. The microbes invade the pocket epithelium and connective tissue, triggering a host response and damaging tissues [1,2]. Traditional scaling and root planing (SRP) is the cornerstone of periodontal therapy, and it aims to eliminate all bacterial plaque load [3].

SRP is an essential periodontal procedure that facilitates the elimination of supragingival and subgingival deposits. The process of scaling involves the thorough removal of plaque, calculus, and stains accumulated on the surface of teeth roots or crowns. In contrast, root planing focuses on the elimination of altered cementum or rough dentin surfaces that are laden with calculus, toxins, or microorganisms, ensuring a smoother and plaque-free root surface [4].

Periodontal pockets are anatomical spaces formed between the gingiva and teeth due to inflammatory processes triggered by host-microbe interactions. In these pockets, the root surfaces become compromised by the presence of a biofilm composed of plaque and calculus, along with the penetration of various pathogenic bacteria and their toxic byproducts, such as bacterial endotoxins, into the cementum. Complete elimination of these detrimental substances is imperative for restoring periodontal tissue, and it is a crucial factor that cannot be effectively addressed solely through traditional mechanical treatment methods. Furthermore, the ability to reach challenging areas like furcations, concavities, grooves, and distal regions of molars is often restricted in traditional SRP, which significantly hinders the thorough instrumentation of these areas. Therefore, alternative approaches beyond conventional therapy may be necessary to thoroughly address these issues and promote successful periodontal healing [5].

Hence, the advancement of innovative approaches for SRP, along with the enhancement of existing mechanical instruments is essential. Due to its ability to achieve effective tissue ablation and exert potent bactericidal and detoxifying properties, laser technology holds great potential as a novel technical option for non-surgical periodontal therapy. Additionally, lasers offer the advantage of accessing areas that are inaccessible by traditional mechanical instruments.

In addition, traditional mechanical therapy typically creates a smear layer, which can hinder the recovery of periodontal tissues due to the presence of bacteria and inflammatory agents like remnants of infected cementum and calculus [6]. In contrast, laser treatment does not result in the formation of a smear layer [7].

Scaling, root planing, and curettage procedures lead to the formation of a long junctional epithelium without connective tissue attachment. [5] Conversely, laser curettage removes the epithelium lining and inflammatory cells from soft tissue pocket walls. The weakened scattered low-energy laser potentially promotes periodontal tissue attachment and reduces postoperative pain by stimulating the surrounding tissue cells [7]. This results in more lymph flow [8], increased cell proliferation [9,10], and reduced inflammation [11-13].

This review aims to offer a thorough comparative overview of various laser therapies in comparison to traditional scaling and root planing, as well as to pinpoint the existing gaps in the literature and investigate the current state of non-surgical laser therapy in the treatment of periodontitis.

## Review

Research methodology

A search was conducted on digitized databases such as PubMed, Embase, Medline, and Google Scholar, utilizing specific keywords such as "lasers," “periodontal therapy," "scaling," and “root planing." After the screening and evaluation process, the resulting articles included narrative reviews, systematic reviews, randomized controlled trials, comparative studies, split-mouth studies, case series, and reports based on non-surgical laser therapy and the use of lasers as an adjunct to SRP.

The criteria for study selection included research on non-surgical periodontal therapy, such as SRP, either in comparison with laser therapy or as an adjunct or monotherapy. Moreover, in-vitro studies utilizing lasers on root surfaces, as well as articles focusing on lasers and non-surgical periodontal therapy, were considered. Conversely, studies involving surgical interventions for periodontitis and the adjunctive use of antimicrobial photodynamic therapies were specifically excluded.

History of lasers

In 1960, physicist Theodore H. Maiman created the forerunner of the laser, known as the “Maser,” in the United States. This device comprised a single crystal rod made of artificial ruby, and it could produce red light with a wavelength of 694 nm within the microwave spectrum. The Maser, also known as Microwave Amplification by Stimulated Emission of Radiation (MASER), is commonly referred to as “laser” in present times. The initial iterations of lasers were referred to as optical masers. The term LASER stands for Light Amplification by Stimulated Emission of Radiation. In 1964, Charles H. Townes was honored with the Nobel Prize for his contribution to the field of laser technology. Townes is credited as the pioneer who successfully strengthened the microwave spectrum of radiation through stimulated emission. The genesis of the laser emanates from Einstein's theoretical framework, which was elucidated in the early 1900s. In 1917, through his Quantum Theory of Radiation, Albert Einstein posited the possibility of stimulating segments of the electromagnetic field to generate intensified light [7,14,15].

The theory of laser action

The illumination energy can initiate energy switching within atoms, prompting the atoms to shift from their ground state to a state of excitement or activation by the absorption of a quantum of energy. [16] This phenomenon is referred to as “stimulated absorption.” Because the state with the lowest energy level is highly stable, the excited atom typically reverts to its normal state by expelling a quantum of energy spontaneously, a process known as “spontaneous emission.” The transition back to a lower energy state can be facilitated by continuously exciting the activated medium by exposing it to a quantum of light with a matching transition frequency, termed “stimulated emission” [17]. Throughout this process, a photon equivalent in size to the emitted atom is released, which then interacts with the neighboring activated atom and triggers a cascade of photon emissions.

Properties of lasers

Laser light possesses distinct characteristics that set it apart from other forms of light. These include being monochromatic, which means it consists of light of a single specific wavelength; exhibiting collimation to ensure beam divergence and parallelism; and being coherent, which involves synchronization of all waves to uphold a consistent phase relationship among each other. These qualities enable highly directional, low divergence, and monochromatic laser beams to be directed onto target tissue in various modes, such as continuous wave, gated-pulse mode, or free-running pulse mode [18,19].

In continuous-wave mode, the laser emits a continuous beam at a constant power level when activated. In gated-pulse mode, the laser alternates between on and off states at specific time intervals, with the duration of each state measured in microseconds. In free-running pulse mode, the laser releases a high amount of energy for a very short period, typically in microseconds, followed by a prolonged period of inactivity. [15] Table 1 shows the properties of the laser used in periodontal therapy.

**Table 1:** Laser therapy in periodontal treatment technical points

Laser Therapy in Periodontal Treatment Technical Points			
ND Lasers	Erbium Lasers	Diode Lasers	CO2 Lasers
Operation mode: Free-running pulsed wave. Fiber optic tip: 400 MM (core diameter: 320 MM). Power settings: optimal at 100 MJ/pulse; avoid higher settings to prevent thermal damage.	Water absorption: high, reducing thermal impact. Power settings: effective at 160 MJ/pulse; avoid exceeding 200 MJ/pulse to prevent damage.	Mode: continuous wave or pulsed. Fiber optic system: contact mode. Power settings: effective at low powers (1-1.5 W)	Mode: pulsed and continuous wave. Soft tissue use: primarily used for soft tissue due to potential thermal damage to hard tissues. Pulsed defocused mode: preferred for root conditioning and bactericidal effects. Caution: use low powers and energy densities to prevent damage to healthy hard tissues.

Types of lasers

Presently, existing laser systems are categorized based on their active medium into various types. These include gas lasers (He: Ne, CO2, excimers, or argon lasers); solid-state lasers (Nd:YAG, holmium-doped yttrium aluminum garnet or Ho:YAG lasers, Er:YAG, erbium, chromium, yttrium scandium gallium garnet or Er,Cr:YSGG lasers, Rubin, and alexandrite lasers); liquid (dye) lasers (which utilize liquid colorants like Rhodamine G6 and Coumarin); and semiconductor (diode) lasers like gallium-arsenide (GaAs) and gallium-aluminum-arsenide (GaAlAs) lasers, which use semiconductors as the medium. Based on their operational mode, lasers are classified as either continuous or pulsed [7,14].

Lasers are categorized based on the wavelengths of light in the electromagnetic spectrum. Excimer lasers such as argon fluoride (193 nm) and xenon chloride (308 nm) fall under the ultraviolet spectrum, while argon and He-Ne lasers are found in the visible spectrum (400-700 nm) [7]. Some lasers emit rays in the far or middle infrared spectrum, such as CO2 lasers (10600 nm), which are hydrophilic, and erbium lasers (Er:YAG = 2940 nm and Er:YSGG = 2790 nm), which have increased affinity for hydroxyapatite. As hydroxyapatite is highly absorbed in water-rich tissues, it leads to the vaporization or evaporation of fluid. Conversely, lasers emitting in the near-infrared spectrum act on tissue macrophages and pigments, in addition to poor water absorption, causing a coagulation effect. These include the Nd:YAG laser (1064 nm) and the diode laser (655-980 nm) [20,21].

Based on the power of the emitted laser energy, lasers can be classified into low-power lasers and high-power lasers. Due to their bio-stimulating effect on tissue cells, low-energy lasers can effectively treat gingivitis and periodontitis by increasing mitotic activity and stabilizing the epithelium, thereby reducing the risk of periodontal disease progression [22]. Conversely, the operational principle of high-energy lasers is centered on causing irreversible effects by vaporizing or evaporating water from tissues. This makes them suitable for various procedures such as gingivectomies, gingivoplasties, frenectomies, tissue biopsies, and the removal of reactive lesions [23]. Table 2 shows different laser applications in periodontal therapy.

**Table 2:** Different laser applications in periodontal therapy SRP: scaling and root planing; PD: probing depth; BOP: bleeding on probing; CAL: clinical attachment level

Laser Therapy			
ND: YAG lasers versus traditional scaling and root planning	Erbium lasers versus traditional scaling and root planning	Diode lasers versus traditional scaling and root planning	Coz lasers versus traditional scaling and root planning
Adjunct to SRP: use ND lasers after initial mechanical debridement. Microbial reduction: effective in reducing SUB-GINGIVAL MICRBES and IL-1B without significant PD and CAL improvements. Optimal settings: use 100 MJ/pulse to avoid thermal damage and ensure efficacy. Potential benefits: useful for microbial disinfection in areas like furcation, though long-term effectiveness is uncertain.	Alternative or adjunct: Erbium lasers can be standalone or supplement SRP. Effective for PD, CAL, and Bop: clear benefits over SRP at 160 MJ/pulse. Power setting caution: avoid exceeding 200 MJ/pulse and below offer no added benefits. Deep pockets: effective for 4-6 MM deeper pockets with proven long-term outcomes.	Adjunct to SRP: use diode lasers with SRP, not alone. Microbial reduction: mitigates microbial growth when used as an adjunct. Optimal settings: use low powers (1-1.5 W) to avoid thermal damage. Moderate PD: effective for managing 4-6 MM pockets.	Soft tissue use: CO2 lasers are mainly for soft tissue to avoid thermal damage to hard tissues. Adjunctive use: use with SRP at low powers to protect healthy hard tissues. Root conditioning: prefer pulsed defocused mode for root conditioning and bactericidal effects. More research: needs long-term studies with larger sample sizes. Are needed to comprehensively analyze the advantages and disadvantages.

Nd:YAG lasers versus traditional scaling and root planing

The Nd:YAG laser operates as a free-running pulsed-wave laser that emits light at a wavelength of 1,064 nm [14]. Its delivery is conveniently facilitated through a flexible optical fiber equipped with a contact tip measuring 400 μm (core diameter: 320 μm), which is well-suited for pocket insertion [7]. In 1997, the FDA approved the use of pulsed Nd:YAG lasers for sulcular debridement [24].

Arcoria and Vitasek-Arcoria [25] evaluated the effects of an Nd:YAG laser on removing calculus from root surfaces in a controlled environment. The Nd:YAG laser treatment was carried out at the interface between calculus and cementum using contact mode at power levels of 1.5 or 3.0 W (15 Hz), with the tip positioned at a 45-degree angle to the root surface. When subjected to 1.5 W of irradiation, there was no significant impact on the attachment between the calculus and the root surface. However, at 3.0 W of irradiation, the calculus was removed without causing damage to the root surface; this was similar to the outcomes achieved through traditional hand instrumentation.

Cobb et al. [26] conducted a study where they utilized Nd:YAG laser irradiation in periodontal pockets in vivo with a power range of 1.75-3.0 W (87.5-150 mJ/pulse, 20 Hz), either independently or in conjunction with manual instrumentation. The use of the Nd:YAG laser led to incomplete calculus removal with thermal damage to the root surface and created a distinct porous calculus surface devoid of microbial plaque. They also evaluated laser therapy followed by mechanical debridement and observed reduced subgingival microflora post-treatment.

Horton and Lin [27] conducted a comparison between the subgingival administration of the Nd:YAG laser and the conventional method of SRP. A total of 15 patients, each with three segments (45 segments in total), were subjected to an Nd:YAG laser treatment (2 W: 100 mJ/pulse and 20 Hz, 2 min), SRP using a curette, or no intervention. The results indicated that subgingival application of the Nd:YAG laser was as effective as, if not more effective than, SRP in preventing the recolonization of specific bacterial strains. While the laser treatment did not remove calculus as efficiently, it demonstrated comparable effectiveness in terms of reducing probing depth (PD) and attachment loss.

Radvar et al. [28] conducted a study using either SRP or an Nd:YAG laser at low power settings of 0.5 or 0.8 W (50 or 80 mJ/pulse, 10 Hz) in 11 patients at 80 sites. The findings of the scanning electron microscope revealed that low levels of Nd:YAG laser energy did not cause thermal damage to the root surface. However, laser treatment did not produce improvements in the clinical and microbiological aspects of periodontal disease compared to SRP.

In a split-mouth randomized controlled clinical experiment, Liu et al. [29] examined the effects of SRP versus Nd:YAG laser therapy (3 W: 150 mJ/pulse and 20 Hz) on crevicular IL-1b levels in eight patients at 52 sites. The results indicated that Nd:YAG laser treatment alone (3 W: 150 mJ/pulse and 20 Hz) was less effective in reducing crevicular IL-1b compared to traditional SRP. Interestingly, laser therapy performed following SRP, after a six-week interval, led to a more significant decrease in IL-1b levels and better clinical outcomes compared to other treatment sequences. However, no additional advantage was observed when laser treatment was given after traditional SRP therapy.

Miyazaki et al. [30] compared Nd:YAG (2.0 W: 100 mJ/pulse and 20 Hz, 120 s) and CO2 laser (2.0 W, 120 s) treatments with ultrasonic scaling in chronic periodontitis patients with periodontal pockets. After treatment, Porphyromonas gingivalis and gingival crevicular fluid significantly decreased in the Nd:YAG and scaling groups. Additionally, all three groups displayed decreased inflammation and PD. While the Nd:YAG group also exhibited a trend toward decreased IL-1 levels, the CO2 laser with 100 mJ power did not effectively remove subgingival plaque.

Abbasi et al. [31] conducted a systematic review and meta-analysis on the use of Nd:YAG laser (1064 nm, 75-100 mJ/pulse, and 60-120 s) therapy with SRP versus standalone SRP for periodontitis. They found a significant reduction in periodontal PD and interleukin 1-beta levels with the laser therapy group. Due to limited research in this area, the debate continues as to whether Nd:YAG laser therapy with SRP is more effective than SRP alone for treating periodontitis.

Next, de Andrade et al. [32] conducted a study examining bacterial reduction in class II furcation defects (34 lesions from 17 patients) in chronic periodontitis patients using Nd:YAG (100 mJ/pulse; 15 Hz; 1.5 W, 60 s, 141.5 J/cm2) laser irradiation combined with SRP (test group) versus SRP alone (control group). The experimental group initially showed the highest reduction in colony-forming units, but this effect was not sustained after six weeks. Both treatments resulted in improvements in clinical parameters, suggesting that Nd:YAG laser combined with conventional therapy significantly decreased the bacterial count in furcation defects, especially in the short term.

In the aforementioned research studies, one study [28] indicated a significant mean decrease in PD in the control group (SRP) compared to the laser-treated group over six weeks. The PD reduction in the laser and control groups was 0.50 mm and 1.70 mm, respectively, while the reduction in bleeding on probing (BOP) was 10% and 45% in the laser and control groups, respectively. Another study [30] reported minimal variations in PD reduction between laser-treated sites (1.43 mm) and control sites (1.36 mm) over 12 weeks, with no notable differences in clinical attachment level (CAL) gain between laser-treated (0.50 mm) sites with control site (0.57 mm). The BOP reduction was 43% and 34% in the laser and control groups, respectively. The studies reviewed in the systematic analysis [31] displayed a notable decrease in PD from baseline to follow-up when comparing laser treatment with SRP and SRP alone. The PD reduction ranged from 1.16-4.2 mm for the laser with the SRP group and 0.84-2.0 mm for the SRP alone group. In another investigation [32], the vertical component of furcation PD reduction after six weeks was minimal, measuring 1.8 mm (test group) and 1.9 mm (control group), with a slight difference in CAL reduction between the two groups. The horizontal PD reduction was 0.6 mm in the test group and 0.9 mm in the control group.

Therefore, based on the evidence provided, it is recommended that Nd:YAG lasers be used as an adjunct to SRP, especially following initial mechanical debridement. They have demonstrated benefits in reducing sub-gingival microbial levels and IL-1b without additional improvements in PD and CAL compared to SRP alone. The ideal power setting for Nd:YAG laser therapy is 100 mJ/pulse to avoid thermal damage at higher settings and to overcome reduced efficacy at lower settings, with potential benefits for microbial disinfection in areas like furcation with uncertain long-term effectiveness. Future long-term and larger sample size research should focus on Nd:YAG laser pocket treatment for deeper probing depths beyond the 4-6.5 mm range seen in the currently discussed studies.

Erbium lasers versus traditional scaling and root planing

In 1974, Zharikov et al. developed the Er:YAG laser [33]. Subsequently, in 1999, the FDA sanctioned the use of the pulsed Er:YAG laser for procedures involving soft tissue and sulcular debridement. Among the lasers that operate within the near- and mid-infrared spectrum, the Er:YAG laser stands out for its strong absorption in water. This is attributed to its 2,940 nm wavelength, which corresponds with a major absorption band of water. This heightened water absorption characteristic of the Er:YAG laser helps minimize the thermal impact on surrounding tissues during treatment [7]. Currently, two distinct erbium laser wavelengths are applied in clinical practice: the Er:YAG (2940 nm) and the Er,Cr:YSGG (2780 nm) [34].

Schwarz et al. [35] conducted a split-mouth randomized controlled trial to compare the efficacy of Er:YAG laser irradiation versus traditional scaling and root planing in the treatment of periodontal pockets. The study involved 20 patients (110 teeth with 660 sites, PD > 4 mm). The Er:YAG laser treatment utilized specific contact tips at a panel setting of 160 mJ/pulse and 10 Hz with water coolant, moving from the coronal to the apical direction in parallel paths at a 15-20 degree inclination to the root surface. This laser treatment was found to be more time-efficient compared to SRP. After a six-month follow-up, results showed that the laser treatment yielded similar or superior outcomes in terms of reduction of bleeding on probing (BOP), PD, and clinical attachment level (CAL). Notably, the laser group demonstrated a significantly greater reduction in BOP and improvement in CAL when compared to the SRP group, especially in deeper pockets. The researchers suggested that the Er:YAG laser could be a viable alternative to conventional SRP. Schwarz et al. [36] further documented that the clinical CAL gain achieved after Er:YAG laser therapy remained stable throughout a two-year duration. Schwarz et al. [37] further examined the requirement for adjunctive SRP following Er:YAG laser therapy. A clinical investigation similar to the previously cited research [35] was conducted, and it revealed a lack of significant enhancement in clinical results with no added advantage when comparing laser therapy alone to laser therapy in combination with SRP.

Crespi et al. [38] performed an in vitro study that combined SRP with an Er:YAG laser at different energy levels, such as 100 mJ/pulse, 200 mJ/pulse, and 250 mJ/pulse. They found that it led to various outcomes, such as thermal damage, fractures, carbonization, and removal of the cementum layer. At 300 mJ/pulse, significant grooves and craters were observed. In an in vivo study, Crespi et al. [39] revealed that the laser tip operating at 140 mJ and 10 Hz introduced some minor irregularities and at 160 mJ and 10 Hz produced a consistent and uniform surface without alterations or thermal burn.

Furthermore, Crespi et al. [40] demonstrated that Er:YAG laser treatment enhances fibroblast attachment on periodontally diseased root surfaces compared to ultrasonic instrumentation. This finding is supported by Hakki et al. [41], who found that Er,Cr:YSGG laser treatment promotes biocompatibility for human PDL fibroblast attachment on diseased root surfaces, while untreated surfaces hinder cell attachment.

Kelbauskiene et al. [42] conducted a study that compared the adjunctive application of Er,Cr:YSGG laser (9 mm Z-6 tip used at a setting of 1 watt) with conventional SRP and SRP alone. The research involved treating ten adult individuals with periodontitis (PD of 4 mm at least on one aspect of the tooth) following a split-mouth design, utilizing either Protocol A (SRP) or Protocol B (Er,Cr:YSGG laser therapy in combination with SRP). The findings illustrated that the non-surgical periodontal intervention, with or without laser therapy, resulted in noteworthy enhancements across all assessed parameters (plaque accumulation, BOP, and PD) three months post-treatment. A more significant decrease in plaque levels was observed in the laser-SRP-treated group. Particularly, the combined approach incorporating laser therapy as an adjunct to SRP exhibited superiority over SRP alone; this was primarily attributed to its enhanced efficacy in restoring CAL.

Lopes et al. [43] conducted a split-mouth study comparing the effects of the Er:YAG laser (100 mJ/pulse, 10 Hz, 12.9 J/cm2) with and without SRP to SRP alone. In this study, 21 participants with PDs ranging between 5 mm and 9 mm in four non-adjacent sites (84 sites) were split into SRP and laser, laser alone, SRP alone, or no intervention groups. A reduction in the plaque index (PI) was observed in groups receiving SRP and laser, as well as SRP alone, 12 days post-treatment. BOP decreased significantly in groups receiving SRP and laser, laser alone, and SRP at 12 days post-treatment. PD also decreased significantly after 30 days in treated groups, with differences noted between the SRP and laser groups compared to the no-treatment group. However, no difference in IL-1β was found among groups. Er:YAG laser could be an adjunctive for the treatment of periodontal pockets, as significant CAL improvement was primarily associated with SRP rather than laser therapy.

Lopes et al. [44] demonstrated that using an Er:YAG laser operating at 100 mJ/pulse and 10 Hz in conjunction with SRP resulted in a notable decrease in levels of Aggregatibacter actinomycetemcomitans (Aa), Porphyromonas gingivalis (Pg), Prevotella intermedia (Pi), Treponema denticola (Td), and Tanerella forsythia (Tf) 12 months post-treatment. The application of only laser therapy led to a decrease in Pg levels. The researchers concluded that the Er:YAG laser has the potential to mitigate and manage the multiplication of microorganisms in patients with persistent periodontitis.

Gurpegui Abud D et al. [45] conducted a split-mouth randomized controlled clinical trial comparing conventional SRP with Er:YAG laser for treating generalized Stages II or III and Grade B periodontitis. They divided 30 subjects into two groups: conventional scaling and root planing (C-SRP) versus laser-assisted scaling and root planing (L-SRP). Both treatments showed improvement without significant differences in clinical outcomes (CAL gain or PD reduction). L-SRP took half the time of C-SRP, but C-SRP had more postoperative sensitivity. The low-energy Er:YAG (50 mJ) protocol used in this study had similar clinical results to conventional scaling and root planing for Stage II-III and Grade B periodontitis.

In their systematic review, Vagdouti et al. [46] examined the prolonged impact of Er:YAG or Er,Cr:YSGG lasers either alone or combined with mechanical therapy. The majority of the studies included in this review used 160 mJ/pulse and 10 Hz lasers. Clinical outcomes such as PD, CAL, BOP, and gingival index (GI) were evaluated in chronic periodontitis patients (the baseline PD of the studies reviewed ranged from 3-8 mm). They found that Er:YAG and Er,Cr:YSGG lasers, as monotherapy or adjunct to SRP, improved CAL and reduced PD in the long term, particularly in deep pockets ≥ 7 mm.

Linhua Ge et al. [47] assessed Er,Cr:YSGG (1.25 W, 30 Hz) laser use for managing root furcation involvements in 30 patients (128 teeth with degree II or III furcation involvement). They adopted a split-mouth design comparing laser treatment (Group A) to manual debridement (Group B) using PD, BOP, CAL, and visual analog scale (VAS) pain scores at six and 12 weeks. Both treatments reduced PD, CAL, and BOP. However, laser treatment showed lower VAS pain scores and significantly higher reductions in PD and BOP compared to manual debridement.

After a collective analysis of the data from the aforementioned erbium laser studies on nonsurgical periodontal therapy, one study [35] revealed an insignificant reduction in mean PD of 2.0 mm (laser group) and 1.60 mm (SRP group) from baseline to six months. Whereas, a significant reduction in mean BOP score in the laser (56%-13%) and control group (52%-23%) and a significant gain in mean CAL were established between the laser (1.9 mm) and control groups (1.0 mm) from baseline to six months. These gains in CAL were maintained and reported in a follow-up study [36] at two years (laser group: 1.4 mm vs. control group: 0.70 mm). Another study [42] showed that three months after treatment, the laser group and the control group had PD reductions of 2.00 mm and 0.97 mm, respectively, with BOP reductions of 68% in the laser group and 60% in the control group. In another study [43], the reduction in PD was more or less equivalent between the laser+SRP (1.60 mm) and the SRP group (1.67 mm), whereas the gain in CAL was 0.21 mm for the laser+SRP group and 0.48 mm for the SRP group. In a clinical trial [45] discussed above, at three months post-treatment, examination of all sites exhibiting PD > 4 mm (≥ 3 mm CAL) revealed a mean reduction of 1.74 mm in PD (mean CAL gain of 0.88 mm) for the SRP group and 1.72 mm in PD reduction (mean CAL gain of 0.86 mm) for the laser group. Further evaluation of all sites with PD > 6 mm (≥ 5 mm CAL) demonstrated a mean PD reduction of 1.85 mm (mean CAL gain of 1.46 mm) in the SRP group and a marginally greater PD reduction of 2.05 mm (mean CAL gain of 1.41 mm) in the laser group. The research studies examined in the systematic review [46] revealed a reduction in PD that varied from 0.52-4.87 mm in the laser group and 0.36-2.29 mm in the control group. The CAL gain ranged from 0.68-5.03 mm in the laser group and 0.15-2.01 mm in the control group. A study [47] compared the Er,Cr:YSGG laser with traditional subgingival instrumentation in Grade II or III furcation involvement with PD ≥ 4 mm. The results showed PD reduction at 12 weeks in both groups (laser group -1.45 mm and control group -0.99 mm), while the CAL gain at 12 weeks in the laser group (1.22 mm) and the control group (1.01 mm) revealed that the laser group had slightly better PD and CAL results. 

The critical evaluation of the data and literature presented above suggests that an erbium laser can act as a standalone treatment or a supplement to SRP. It demonstrates clear benefits over SRP when utilized at appropriate power levels of 160 mJ/pulse in terms of PD, CAL, and BOP. However, excessive power exceeding 200 mJ/pulse may result in thermal damage. Conversely, lower power settings at 100 mJ/pulse and below do not offer additional benefits. Lower power levels also appear to be less effective compared to SRP alone, although they do exert bactericidal disinfection effects. Furthermore, erbium laser therapy has been proven to be effective in treating moderate (4-6 mm) and deeper pocket depths (7 mm or more) with proven long-term outcomes at suitable power settings (160 mJ/pulse). While erbium lasers show promise in reducing pocket depths, their efficacy in areas like furcation remains uncertain over the long term. Hence, erbium lasers are a viable alternative to traditional SRP.

CO2 laser versus traditional scaling and root planing

The CO2 laser, operating at a wavelength of 10,600 nm, functions as both a pulsed and CW laser by employing a blend of 4.5% CO2, 13.5% nitrogen, and 82% helium in specific proportions as its active medium [14]. As the CW CO2 laser is known to cause thermal damage such as cracking and carbonization on hard tissues, it is mainly utilized for soft tissue procedures [48-50]. Despite having a one-tenth water absorption coefficient compared to the Er:YAG laser, caution is advised during subgingival periodontal therapy with the CO2 laser to prevent damaging healthy hard tissues. Low power and energy densities should be meticulously employed. Furthermore, the introduction of the super-pulse mode has helped reduce potential adverse effects from excessive heat generation, and advancements from conventional delivery systems (mirror systems with articulated arms) to modern systems such as the development of flexible fiber optic delivery may lead to broader applications of the CO2 laser in periodontal pocket treatment [7,34].

Crepsi et al. [51] performed an in vitro investigation on periodontally compromised teeth that were extracted by utilizing the CO2 laser in pulsed defocus mode at 2 W and 1 Hz. A total of 30 single-rooted human teeth were extracted (60 specimens collected from all teeth chosen) and divided into three groups: control (untreated), hand SRP, and laser (CO2 pulsed defocused) with ultrasonic scaling. The group treated with laser and scaling exhibited the highest quantity of firmly attached fibroblasts compared to the untreated control group and SRP alone. The researchers therefore deduced that the application of pulsed defocus mode CO2 laser treatment in conjunction with mechanical instrumentation is a valuable approach for root conditioning.

In a split-mouth study, Pope JD et al. [52] evaluated the efficacy of a CO2 laser (8-W pulsed mode, 0.8-mm spot size, energy density of 150-250 mJ/cm2) de-epithelialization method in preventing epithelial down growth in treatment for severe, chronic periodontitis when combined with SRP (test sites) as opposed to SRP alone (control sites). Although there was a tendency for sites treated with the CO2 laser to have lower PD, better CAL, and greater recession, the results did not show statistically significant improvement over SRP alone.

Coffelt et al. [53], in an in vitro study, observed that the CO2 laser eliminated microbial colonies with minimal damage to root surfaces when operated in the defocused mode at an energy density of 11-41 mJ/cm2.

Similarly, Everett JD and colleagues [54] conducted a split-mouth study to investigate the benefits of adding CO2 laser (1st pass: 25-50 J/cm2 at 4 watts CW and 2nd pass: 75-100 J/cm2 at 8 watts CW) treatment for sulcular decontamination to SRP in the management of chronic periodontitis. The research involved 173 teeth from 14 participants. They were separated into two groups: a group that underwent SRP with CO2 laser therapy and a group that only received SRP. Outcomes revealed that sites treated with laser therapy showed a greater decrease in PD, an improvement in CAL, and reduced bacterial levels. In conclusion, the results imply a potential advantage of combining laser therapy with SRP for the management of chronic periodontitis.

In the study [52] discussed above, a decrease of 2 mm in PD was observed at the test site (resulting in a 1.3 mm gain in CAL). Whereas, at the control site, the decrease in PD was 1.5 mm (0.8 mm CAL gain) for all sites with PD equal to or greater than 5.0 mm after three months. The reduction in BOP was 42% at the test site and 37% at the control site. In another study [54], for all sites having PD of 5.0 mm or higher after three months, the PD decrease was 1.73 mm (1.50 mm CAL gain) in the test site and 1.58 mm (1.21 mm CAL gain) in the control site. In contrast, the PD decrease was 2.02 mm (1.83 mm CAL gain) at the test site and 1.42 mm (1.44 mm CAL gain) at the control site for all sites with PD of 5.0 mm or above at six months. Thus, the pulsed defocused mode is considered more favorable than the CW mode when utilizing a CO2 laser due to its superior benefits, such as root conditioning effects, bactericidal properties, and ability to reduce thermal damage. Despite the slightly better performance of PD reduction with CO2 laser compared to SRP, further extensive long-term studies with large sample sizes and greater probing depths are needed to comprehensively analyze the advantages and disadvantages of CO2 laser, as current studies on this topic are limited.

Diode laser versus traditional scaling and root planing

The GaAlAs laser operating at 810 nm and the indium-gallium-arsenide (InGaAs) laser operating at 980 nm are the most commonly used diode lasers [34]. These semiconductor lasers convert electrical energy to light energy within the 800-980 nm wavelength range. They emit light in CW or gated-pulsed modes and are delivered through a fiber optic system in contact mode. The lasers have high absorption rates in hemoglobin and other pigments but low absorption rates in water. In 1998, the Food and Drug Administration approved the use of diode laser GaAlAs with a wavelength of 810 nm for sulcular debridement [7].

Kreisler et al. [55] conducted an in vitro investigation to analyze the attachment of periodontal ligament cells to root surfaces treated with an 810 nm diode laser. Following SRP of the diseased root surface using curettes and air-powder abrasive treatment, the laser group underwent diode laser exposure at 1 W in CW mode for 20 seconds, while the control group did not receive any irradiation. The results showed no significant disparity in cell attachment between the laser and control groups, indicating that the diode laser did not have any adverse effects on the root surface. Kreisler et al. [56] conducted a study to assess changes in root surfaces when exposed to GaAlAs-diode lasers (809 nm, 0.5-2.5 W, CW, 10-30 s). They found severe damage on blood-coated specimens but minimal effects on dry or saline-moistened specimens. In addition, they observed that irradiation at 1 W or lower had little impact, while irradiation at 1.5 W and above led to partial or complete carbonization. In a related study, Kreisler et al. [57] investigated temperature increases in the pulp during diode laser (809 nm GaAlAs and 0.5-2.5 W in the CW mode for 120 s) irradiation on root surfaces, noting temperature elevations between 0.5 and 32.0°C based on energy and time factors. In an investigation using GaAlAs diode laser therapy on periodontally diseased roots, Schwarz et al. [58] found that the diode laser (1.8 W, pulsed at 810 nm) was ineffective in removing calculus. It also altered the root surface in an undesired way, particularly when high energy levels and CW were used in the presence of blood and at high temperatures.

In a randomized clinical trial conducted by Dukić W et al. [59] , the efficacy of incorporating a 980-nm diode laser (peak power of 2 W and average power of 0.66 W) in conjunction with SRP for the treatment of chronic periodontitis was evaluated in 35 patients. The study followed a split-mouth design, with SRP being performed in two control quadrants (control groups) while the diode laser was utilized adjunctively with SRP in contralateral quadrants (laser groups). Findings indicated that although both control and laser groups demonstrated comparable results across various clinical parameters, the laser group, particularly with multiple adjunctive applications of a 980-nm diode laser, displayed a notable improvement in PD for moderate pockets (4-6 mm) when compared to standalone SRP treatment.

The effect of 980 nm diode laser (1.5 W in pulse mode, 20 seconds) treatment combined with SRP on type II diabetes and chronic periodontitis was investigated in a study done by Lalli AK et al. [60]. For the split-mouth research, participants were separated into two groups. The control group had only mechanical debridement, and the test group received laser treatment in addition to mechanical debridement. The test group's GI and PI scores improved, but their CAL levels and PD did not significantly change. Nonetheless, the test group's results outperformed those of the control group. The authors concluded that diode laser in conjunction with non-surgical periodontal therapy reduces PI and GI scores, PD, and increases CAL gains. It also effectively manages chronic periodontitis in patients with type II diabetes.

Kamma JJ et al. [61] conducted a study to compare the impact of SRP, diode laser treatment (LAS) alone, and SRP+LAS in aggressive periodontitis patients and assessed the microbial levels and clinical parameters. They used a 980-nm diode laser at 2 W power (continuous focused mode) for 30 seconds. Bacterial counts decreased in all groups post-treatment and did not revert to baseline levels after six months, with SRP + LAS showing the most significant reduction in total bacterial count, Pg, and Td at six months post-treatment compared to other treated groups. Differences in PD and CAL were noted between the SRP+LAS group and the other treated groups at the six-month mark. No variations were observed in PI and BOP percentages among the treatment groups post-six months. The study suggests that SRP+LAS is more effective in treating aggressive periodontitis compared to SRP or LAS alone over six months.

Qadri et al. [62] performed a systematic review on the impact of adjunctive diode laser treatment with SRP in chronic periodontitis. They found that in patients with PD ≤ 5 mm, the combination of SRP and diode laser (808-980 nm) was more effective than SRP alone for treating chronic periodontitis. 

When addressing the analysis and appraisal of the aforementioned studies, one research study [59] revealed a mean PD reduction of 1.49 mm (1.59 mm CAL gain) in the control group and 1.68 mm (1.61 mm CAL gain) in the laser group at sites with moderate PD (4-6 mm) from baseline to 18 weeks. Whereas, sites with deep PD (7-10 mm) from baseline to 18 weeks showed a mean PD reduction of 3.57 mm (3.25 mm CAL gain) in the control group and 4.00 mm (3.26 mm CAL gain) in the laser group. Another study [60] demonstrated a PD reduction of 1.85 mm (1.20 mm CAL gain) in the control group and 2.20 mm (1.50 mm CAL gain) in the laser group from baseline to three months at sites with pocket depths of ≤ 5 mm. Another study [61] showed a PD reduction of 2.8 mm in SRP+laser, 2 mm in laser alone, and 2.34 mm in the SRP alone group from baseline to six months at sites with a mean PD of 6.3±0.3 mm. Therefore, the diode laser is found to be effective in the management of moderate PD of 4-6 mm. Importantly, it should be used as an adjunct to SRP, not as monotherapy, and at low powers of between 1-1.5 W to avoid thermal damage. Following mechanical debridement or saline irrigation of inflamed and bleeding pockets, diode lasers can be applied to prevent inadvertent thermal injury caused by the laser's interaction with blood pigments. Diode laser alone was ineffective for calculus removal, but it mitigates microbial growth when used as an adjunct. Future research should investigate the effects of diode laser treatment on larger sample sizes and deeper probing depths. Table 3 shows a summary of the comparison of laser types.

**Table 3:** Summary comparison of laser types versus traditional scaling and root planing SRP: scaling and root planing; PD: probing depth; BOP: bleeding on probing; CAL: clinical attachment level

Laser Type	Wavelength	Key Studies and Authors	Settings	Findings	Comparison to SRP
Nd:YAG Laser	1064 nm	Arcoria and Vitasek Arcoria. [25], Cobb et al. [26], Horton and Lin. [27], Radvar et al. [28], Liu et al. [29], Miyazaki et al. [30], Abbasi et al. [31], de Andrade et al. [32]	100 mJ/pulse	Effective in reducing subgingival microflora and inflammation, incomplete calculus removal, the thermal damage at higher settings	Comparable or slightly better than SRP in reducing probing depth (PD) and attachment loss (AL), best used as an adjunct to SRP
Erbium Lasers (Er:YAG, Er,Cr:YSGG)	2940 nm, 2780 nm	Schwarz et al. [35-37], Crespi et al. [38-40], Hakki et al. [41], Kelbauskiene et al. [42], Lopes et al. [43-44], Gurpegui Abud D et al. [45], Vagdouti et al. [46], Linhua Ge et al. [47]	160 mJ/pulse	Minimal thermal damage, effective in fibroblast attachment, a significant reduction in PD, BOP, and CAL	Superior to SRP in deeper pockets and in maintaining long-term outcomes
CO2 Laser	10600 nm	Crepsi et al. [51], Pope JD et al. [52], Coffelt et al. [53], Everett JD et al. [54]	8-W pulsed mode	Minimal damage in defocused mode, effective in microbial elimination, reduced thermal damage in pulsed mode	Slightly better PD reduction compared to SRP, more research is needed for long-term effectiveness
Diode Laser (GaAlAs, InGaAs)	810 nm, 980 nm	Kreisler et al. [55-57], Schwarz et al. [58], Dukić W et al. [59] , Lalli AK et al. [60], Kamma JJ et al. [61], Qadri et al. [62]	1-1.5 W	High absorption in pigments, effective in reducing microbial levels, ineffective in calculus removal	Effective in moderate PD reduction when used with SRP, best as an adjunct to SRP at low power settings

## Conclusions

The analysis of the multiple studies reviewed above suggests that erbium lasers have potential as a non-surgical periodontal therapy option over traditional methods. Research reveals that erbium lasers with energy levels of 160 mJ at 10 Hz can improve clinical outcomes regarding PD and CAL, with higher energy levels at 300 mJ causing adverse effects such as grooves and craters. Erbium lasers are effective in controlling microorganism growth and can be used as a singular therapy or in combination with SRP. Furthermore, erbium laser therapy is effective for treating moderate (4-6 mm) and deeper pocket depths (7 mm or more) with long-term outcomes. In contrast, Nd:YAG lasers are unable to entirely remove calculus and can induce thermal damage. To prevent thermal damage at high settings and counteract reduced efficiency at low settings, the ideal power level for Nd:YAG laser therapy is 100 mJ/pulse. It produces similar results to SRP in minimizing attachment loss and PD and has no added advantage at sites with PD of 4-6.5 mm, with ongoing debate on its superiority. Hence, Nd:YAG laser therapy is used as an adjunct to SRP as it mitigates subgingival microbial levels and IL-1 levels. When CO2 lasers are used with high-energy output in CW mode, they are not suitable for calculus removal and root surface debridement due to significant thermal side effects. However, using CO2 lasers with low-energy output in a pulsed or defocused mode can result in root conditioning and bactericidal effects on contaminated root surfaces, making it a valuable approach for root conditioning when combined with mechanical instrumentation. Clinicians utilizing CO2 lasers to treat periodontitis should therefore be aware of safety concerns and anticipate minimal clinical benefits due to the absence of robust clinical trials. Next, diode lasers are poor at removing calculus and can cause detrimental surface changes to the roots. The reason for this inefficiency may be traced back to the possibility of overheating when pigmented deposits interact with diode lasers. Lower power levels of 1 W have little effect, whereas power levels of 1.5 W and greater can cause complete or partial carbonization. Diode laser treatment with SRP eliminates bacteria and promotes periodontal repair. Studies have indicated that SRP combined with a diode laser is more efficient than either procedure alone, as it is effective for treating moderate PD of 4-6 mm. Since there is a lack of evidence on the topic, the question of whether diode laser treatment is superior for managing periodontitis remains unanswered. Therefore, diode lasers can be considered an adjunct but not an alternative. The current study has limitations in comparing the efficiency of different lasers for microbial mitigation. This is due to the varying methods employed in microbiological assessment in each laser study and the lack of standardization in microbial count units across studies.
